# The importance of self-management for better treatment outcomes for HIV patients in a low-income setting: perspectives of HIV experts and service providers

**DOI:** 10.1186/s12981-024-00612-9

**Published:** 2024-05-04

**Authors:** Tegene Legese Dadi, Yadessa Tegene, Nienke Vollebregt, Girmay Medhin, Mark Spigt

**Affiliations:** 1 School of Public Health, College of Medicine & Health Science,, Hawassa University, Hawassa, Ethiopia; 2 School CAPHRI, Care and Public Health Research Institute, Maastricht University, Maastricht, The Netherlands; 3 Department of Epidemiology, Rijksuniversiteit Groningen, Groningen, The Netherlands; 4 Aklilu Lemma Institute of Pathobiology, Addis Ababa University, Addis Ababa, Ethiopia; 5 MERQ Consultancy PLC, Addis Ababa, Ethiopia; 6 General Practice Research Unit, Department of Community Medicine, The Arctic University of Tromsø, Tromsø, Norway

**Keywords:** Self-management, HIV/AIDS care, Perspectives on self-management, Low-income settings

## Abstract

**Background:**

Self-management is the most important strategy to improve quality of life in patients with a chronic disease. Despite the increasing number of people living with HIV (PLWH) in low-income countries, very little research on self-management is conducted in this setting. The aim of this research is to understand the perspectives of service providers and experts on the importance of self-management for PLWH.

**Methods:**

A systematizing expert interview type of qualitative methodology was used to gain the perspectives of experts and service providers. The study participants had experience in researching, managing, or providing HIV service in east and southern African (ESA) countries. All the interviews were audio recorded, transcribed, and translated to English. The quality of the transcripts was ensured by randomly checking the texts against the audio record. A thematic analysis approach supported by Atlas TI version 9 software.

**Result:**

PLWH face a variety of multi-dimensional problems thematized under contextual and process dimensions. The problems identified under the contextual dimension include disease-specific, facility-related, and social environment-related. Problems with individual origin, such as ignorance, outweighing beliefs over scientific issues, low self-esteem, and a lack of social support, were mostly highlighted under the process dimensions. Those problems have a deleterious impact on self-management, treatment outcomes, and the quality of life of PLWH. Low self-management is also a result of professional-centered service delivery in healthcare facilities and health service providers’ incapacity to comprehend a patient’s need beyond the medical concerns. Participants in the study asserted that patients have a significant stake in enhancing treatment results and quality of life through enhancing self-management.

**Conclusion and recommendation:**

HIV patients face multifaceted problems beyond their medical issues. The success of medical treatment for HIV is strongly contingent upon patients’ self-management practices and the supportive roles of their family, society, and health service providers. The development and integration of self-management practices into clinical care will benefit patients, their families, and the health system.

## Introduction

Human immuno deficiency virus (HIV) is a global pandemic, with 39 million people living with HIV and 1.3 million new HIV infections occurring annually [1]. It is one of the leading causes of mortality in the world with over 630,000 deaths and more than 47 million disability adjusted life years (DALYs) every year [1, 2]. HIV severely affects developing countries; in Africa, over 68% of HIV-positive individuals live and more than two-thirds of HIV-related deaths occur [1, 3]. HIV results in a severe form of low quality of life because of social alienation, a lack of support, and fear of rejection, mainly because of discrimination and internal and external stigma [4–7]. The cumulative negative effects of these conditions are poor antiretroviral therapy (ART treatment) outcomes, and increased transmission of HIV [8, 9]. The chronicity of the disease also has a negative impact on the healthcare system of developing countries, because of an ever-increasing caseload of patients on top of already vulnerable health-care systems [10, 11].

The impacts of HIV should be mitigated through better care and support, patient empowerment to take responsibility for their own care, providing patient centered care, and self-management (engaging in healthful behavior) in order to enhance treatment outcomes, quality of life, and to prevent new HIV infections [12–14]. Self-management is one of the most important strategies to address management of minor symptoms, physical, and physiological consequences of HIV [15]. Self-management has been shown to be effective in improving treatment outcome of chronic diseases [16, 17]. It improves the treatment outcome of medical care by empowering patients to take responsibility for their own care [18–20]. Furthermore, it is more effective than usual care to reduce hospitalizations and frequency of facility visits [18, 21]. Self-management is effective because it combines psychological interventions, such as emotional management, life skill lessons, and mindfulness, with medical interventions for the maximal functioning of the patient [22, 23].

Despite the importance of self-management, it has not been widely used in a structured way, and it is not clear how self-management interventions can be optimized, even in countries where it is implemented [24, 25]. In contrast to the context of developed countries, the use of self-management intervention is uncommon in developing countries [18, 26]. In Ethiopia, for example, structured self-management by HIV-positive patients is almost nonexistent, aside from routine daily life practices. Moreover, health service providers do not follow service delivery approaches that empower patients [27, 28]. The HIV prevention, care and treatment guideline and the national strategic plan of HIV in Ethiopia do not consider self-management as an intervention plan [29, 30].

Self-management should be at the forefront of service delivery for the benefit of HIV patients and the health system in developing countries [10, 31]. Thus, it is crucial to acquire a deeper understanding of self-management from the perspective of experts, health service providers, and people living with HIV (PLWH) to develop, test, and incorporate relevant self-management tools and interventions into service delivery. The results will then lay the foundation for further research and the development of self-management interventions. Therefore, the aim of this study is to assess the perspectives of experts and health service providers on why self-management (SM) is important and to identify elements of SM for better treatment outcomes for HIV patients in Sub-Saharan Africa.

## Methods

### Study design

Systematizing expert interview type of qualitative research methodology was used to collect data on perspectives of experts and service providers on self-management among HIV patients. Systematizing expert interview is used to gain access to exclusive knowledge and views possessed by expert on specific issues [32].

### Context and areas of the study

The study focused on east and southern African (ESA) countries, as compared to other regions ESA have the highest concentrations of adults living with HIV, high proportion of new HIV infection, high deaths and DALY loss due to HIV/AIDS as compared to other regions [1, 2]. Those burdens are on top of the weak and fragile health system that lacks adequate and trained human resources [11]. Out of the countries in the ESA, South Africa, Ethiopia, and Uganda were selected for this assessment due to the widespread presence of many of the problems described above.

### Study participants and recruitment procedures

We recruited study participants with a range of experience in researching, HIV program implementation and providing HIV care services in developing countries namely experts from ESA. We used our network in Ethiopia and tried to reach out to international researchers through publications on self-management in low-income countries mainly using email and phone call. The study participants were mainly from three types of expertise: (1) experts involved in HIV and self-management research, which are identified by reviewing papers published on areas of HIV self-management in Africa; (2) experts who have experience in health program areas mainly on HIV with non-governmental organizations, and (3) health care professionals who are working in health facilities. Most of the participants stayed in their respective expertise areas for more than 10 years.

### Sample size

Twenty-four experts and service providers from Ethiopia, South Africa and Uganda were approached through email and phone call. They had experience of program areas and working in NGOs during recruitment, working as researchers and service providers who are nurses and doctors in health facilities (Table 1).

**Table 1:** Study participants, their experience and country of experience

	No. of experts	Country
Experts – in research about HIV and self-management	10	South Africa, Uganda, and Ethiopia
Experts in HIV program areas	8	South Africa, Uganda, and Ethiopia
Health care providers	6	Ethiopia

### Topic guides, data collection and management

The expert interview topic guide was prepared based on the objective of the study, and then substantial improvements were made after a thorough literature review. The main sections of the topic guides were experience of respondents related to HIV; challenges with respect to care and support to HIV patients – individual, communal and health system related challenges, importance of self-management, and practices to promote self-management among service providers. The interviews were conducted by the study team members (TLD, NV, YT and MS). All interviews were conducted through zoom and face to face interview and audio recorded and notes were taken. The mode of interviews was in English with experts in South Africa and Amharic with experts from Ethiopia. The English audio records were transcribed by the study team members. The Amharic audio records were transcribed and translated to English by experienced transcribers and translators. The study team members ensured the quality of transcripts by randomly checking the texts against the audio record at random timeline.

### Data analysis and reporting model

A thematic approach was used to analyze the qualitative data which was supported by Atlas TI version 9 software. During the thematic analysis, we followed the steps described by Braun V and Clarke V [33]. Before coding, researchers familiarized themselves with the data by reading and re-reading textual data and by listening to audio-recordings. After a good understanding of the concepts, the primary codes were developed from the data (i.e., data driven), after which a codebook was developed. Study team members (TLD, YT) independently carried out line-by-line coding of the transcripts. The coded data were reviewed to identify areas of similarity and overlap. The related codes were combined into overarching sub-themes and themes. Then the sub-themes and themes were reviewed against the coded data and the entire dataset to check whether they were in line with objectives of the study.

**Fig. 1:**
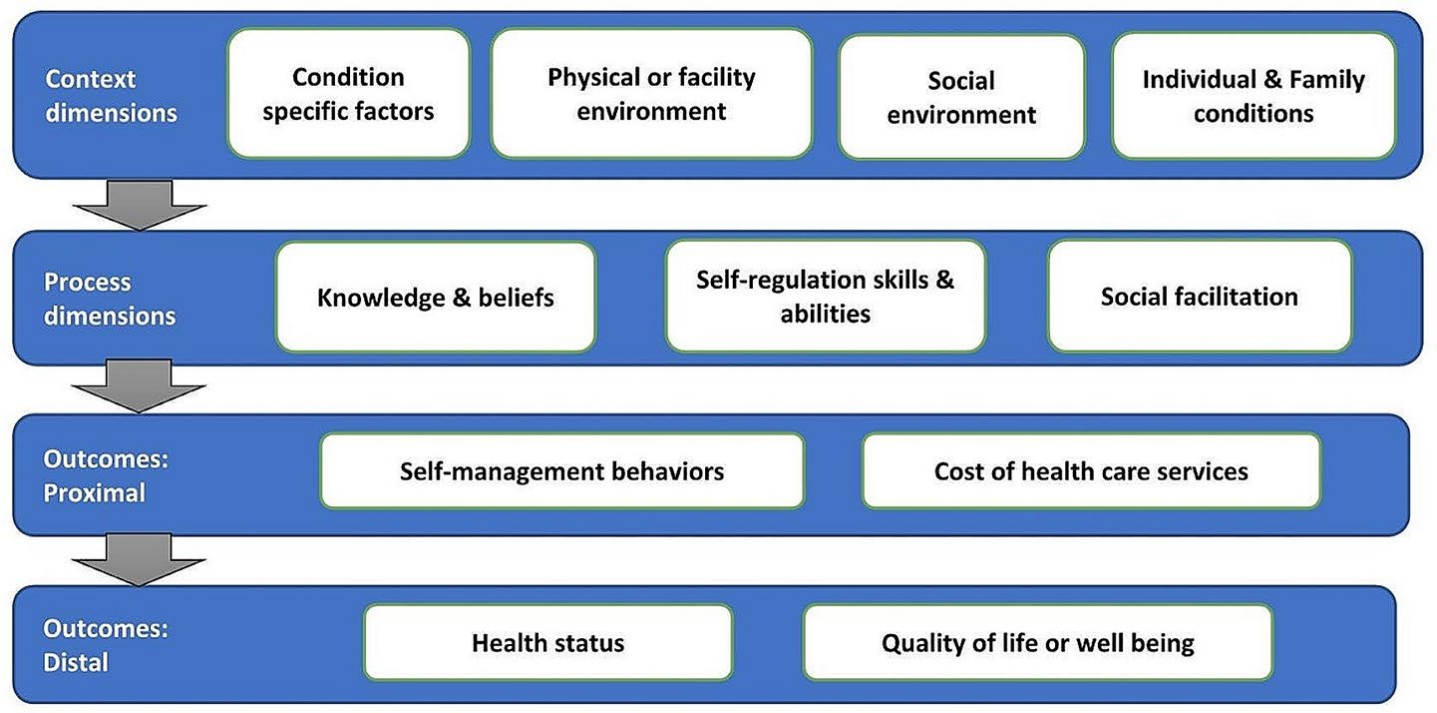
Model of the individual and family self-management theory

In addition, themes were checked to see whether they were clearly and concisely defined with an informative name, and quotes from a range of participants were selected to illustrate themes. The themes and sub-themes were further organized and described using Individual and Family Self-management Theory (IFSMT). This theory is comprehensive that encompasses all dimensions of SM. It includes most chronic care theories and models including the chronic care model. We have arranged the themes in four major dimensions: context, process, proximal outcome and distal outcome using IFSMT with minor modification on the sub-dimensions [23] (Fig. 1).

## Results

We have contacted more than 40 experts and service providers through emails or phone calls; however, only 24 of them have responded to us. Among the total of 24 experts and service providers who responded to our requests, five potential respondents from Uganda declined the interview. Among those experts who declined the interview, three were researchers and two were experts in program areas. Data collected from total of 19 experts (seven experts in research, six experts in HIV program areas and six experts in health care provision) were included in the current study. Four of the study participants were females, and 12 had more than 10 years of experience. Findings are organized into three main themes: context, process and outcome dimensions using the model of the individual and family self-management theory (Fig. 1).

## Context dimensions

### Condition specific factors: lack of understanding that HIV is a lifelong disease

Well-informed HIV patients know that their sickness is lifelong and that it requires lifelong treatment for a better quality of life. Experts say that lacking such understanding makes some patients stop their medication when they feel well. Experts stressed the importance of knowing one’s own status, as well as their own strengths and weaknesses, during the lengthy course of treatment. Patients should be empowered to be responsible and accountable for their own care to achieve a better health outcome. The following quote elaborates on this concept:

In the first place, HIV patients should accept that HIV is a life-long illness that needs treatment and care for life regardless of their clinical condition. Most of our patients are not very well-oriented about this. Sometimes we see patients discontinuing their treatment when they get better. Nurse, service provider, Ethiopia.

### Physical or facility environment: knowledge of health care workers and their relationship with their patients

Experts raised a variety of problems that HIV patients face during the course of lifelong treatment with respect to health facilities. The influence of physical or facility environment on the patient, specifically, the service delivery approach of the health facilities, on the treatment course is high. Most of the experts suggested that the care should be delivered in a patient-centered approach with very good encounters with the patient. Medical care alone will not result in a better treatment outcome; the psychosocial aspect should also be included in the treatment package. The following quote signifies the findings.

Care should be viewed in a broader context rather than only focusing on clinical treatment. You know that, in addition to appropriate and effective clinical medication, adequate psychological support and good interactions with patients are critical for treatment success. If I received better psychosocial assistance in addition to clinical medications, I would be more motivated to take the prescribed medications. So, remembering that everything I do as a health care professional is for a person, putting the patient in the middle and dealing with the problems is crucial. Researcher, University of the Western Cape, South Africa.

Providers should improve the resilience of the patient towards stigma and discrimination to improve the disclosure status of the patient. Experts said that patients are more amenable to disclose when they are resilient. Resilient patients can be more open and figure out who to reveal to and to participate in more stigma-reduction efforts. To boost the quality of life of HIV patients, healthcare professionals should know how to equip and empower their patients, i.e., their self-management skills. Besides helping them to increase their quality of life, it also has a positive impact on reducing the caseload of health facilities. One of the experts from South Africa said that they are using self-management as part of service delivery to reduce caseload from health system and to improve quality of life of patients. The following quotes depict the above evidence.

In South Africa, in some health facilities, due to the high number of patients, we are using self-management as part of service delivery. What we do is equip them [PLWH] with the skills of self-management. … So, since these patients are only screened for symptoms and get viral load done once a year, the rest of the time, they must basically care for themselves using their skills of self-management. … we really don’t have the capacity to really have detailed management of patients, so the patient needs to be empowered to care for themselves. Researcher, Stellenbosch University, South Africa.

Most of the experts described that during service delivery, health professionals should avoid a judgmental approach and instead interact with patients in a friendly and supportive manner and demonstrate emotional intelligence. Knowing the fundamentals of self-management allows health care professionals to be more emotionally intelligent during service delivery. Those skills will help the providers to pick up on what is going on with the client. One of the experts said the following.

…Health professionals should demonstrate emotional intelligence and interact in a supportive manner to their patients to detect what is going on with them when they are having a bad day. Even though the patient did not comply with the care, they [health professionals] must actually say to their patient, “I am not angry with you, I just want to help you,” and the patient is more likely to calm down and engage in a better way. Researcher, Stellenbosch University, South Africa.

### Social environment: disclosure, stigma and discrimination and consequences

It is critical for HIV-positive individuals to inform close family members or friends of their status. It helps patients to easily solicit support from their peers, such as different kinds of support, including psychological support. Moreover, disclosure helps to avoid the fear of stigma and discrimination. However, most patients fear disclosing and then being discriminated. For example, most patients fear attending nearby health facilities, and they travel a very long distance to get the service to avoid meeting people they know. When they feel that they can be discovered by individuals who know them, they discontinue follow-up and go to another facility to get service. Traveling long distances exposes them to a huge amount of expenses, increasing their out-of-pocket expenditure. Furthermore, it is common to see poor adherence among these patients as the result of treatment discontinuation due to frequent changes of facilities. As per the description of one of the experts, those patients do not even ask for referrals when they discontinued and shifted to other facilities again and again, which is contributing to increasing resistance to ART medications.

Most of our patients come from distant areas, some of them from different regions of Ethiopia, and when we tell them to seek service from the nearby health facilities, they are unwilling because of a fear of stigma and discrimination. The problem is that it is common to see treatment discontinuation and poor adherence among these patients because they cannot afford transportation costs for every appointment. To resolve this problem, it is better to work on stigma and discrimination while also encouraging patients to disclose their status. Physician, health service provider, Ethiopia.

Lack of disclosure among adolescents and young people as the result of fear of stigma and discrimination is also contributing to increased transmission of HIV. The following quote demonstrates the effect.

Usually, the difficulty is with females and younger patients. Currently, there are some adolescents who are getting HIV from their families. …, they make sexual intercourse with their peers, and when they join another, they do not disclose sexual activity to their peers, even if there is some. They refrain from disclosure. Researcher, Hawassa University, Ethiopia.

### Stigmas and defense mechanism of patients and solution

The experts identified two types of stigmas: internal and external stigma. The internal stigma can be self-stigmatization; the patient might consider himself/herself worthless and have difficulty socializing with other people. External stigma is the common one, and it is described as fearful society. People have different ways of tackling stigma based on their personality. If someone faces external stigma, he may respond with aggression or self-isolation, substance use or addiction and other reactions. Others could be vulnerable to anxiety and depression. In the case of internal stigma, the situation might be very challenging since it is difficult for the patients to recognize the presence of the problem, evaluate and manage the problems. If it happens, it might be very difficult for the patient to handle by himself/herself. Boosting patients’ skills of self-care or self-management is important to managing emotions and stigma, particularly internal stigma, to prevent depression and other problems and improve the treatment outcome.

It could be difficult in cases of internal stigma. It may be difficult for the client [patient] to evaluate himself or herself, prepare, and fix things. He or she can manage the situation easily if someone who has had the same experience as him or her shares their experience on how to manage the situation. Program expert, NGOs, Ethiopia.

When someone faces external stigma, there are personality differences from one person to another in how they respond to it. … There are individuals who are vulnerable to anxiety and depression; others are vulnerable to other problems. If the situation arose, the client [patient] should assess himself or herself, be aware, and be prepared to respond. Program expert, NGOs, Ethiopia.

### Individual health problems and family support

#### Unhealthy lifestyle: risky behaviors

Experts said that substance use behaviors such as chewing khat, smoking, drug use, and alcohol drinking affect the health status of HIV patients. Use of those substances will affect the stability of patients, such as medication adherence, viral load control, and the occurrence of opportunistic infections. The mental stability of patients can be seen by their adherence to the medication. Besides adherence, stability can also help the patient avoid risky behaviors such as taking drugs, chewing khat, cigarette smoking, and drinking alcohol. The experts suggested combating substance use, as part of improving the quality of life of patients.

Following diagnosis, most HIV patients are not stable and engage in bad behaviors like smoking tobacco chewing khat, drug use, and alcohol drinking. These behaviors have negative consequences, resulting in not starting medication, forgetting to take the medications, and expose other people to the virus. Reducing these behaviors might be difficult to do immediately, but I think they should be reduced step by step and removed if possible. Expert, from government health bureau, Ethiopia.

### Context-specific nutrition

All experts raised the importance of nutrition in improving the status of the patient’s health status. Their feeding should be structured enough to have a balanced diet to cope with the effects of the disease and medication they are taking, which has a high catabolic rate. And thus, the patients should be given or coached by their service providers to prepare their own balanced nutritious menu, which balances all essential macro and micronutrients. While coaching or counseling about nutrition, contextual understanding of the patients’ living status, such as economy and culture is important. As the experts said, it is not uncommon to see service providers recommend their patients to eat expensive food. The experts recommended that the health care professionals should coach or counsel their patients on nutrition, considering the availability in the area and based on their economic level.

...professionals should be considerate of patients’ setting and backgrounds while counselling on nutrition. For example, if the context is in the Gurage area [one of the areas in the southern part of Ethiopia], the nutritious menu can be prepared by making kocho [a food with high fiber prepared from false banana] as the main food item. If there is an option for food or if there is an economic problem, the client can use this and adjust himself/herself. Program person from NGO, Ethiopia.

### Support of families or friends

Families or friends, or individuals around the patient are very important to better treatment outcomes. They can provide support like reminders, material support, assess patient condition, and they can reduce further burdens. A well-supported patient receives good treatment and, as a result, becomes self-reliant and productive, relieving additional burdens from those who support him or her. The lack of support from family or friends has a negative impact on society, or community. It affects the productivity of organizations because of absenteeism and others.

…If I am infected with HIV and living with my family, the burden of the condition is not just on me; it also goes to my family. If I have good support, then I will be able to save money, and the family will be able to save money too. But if I didn’t have good support and if I didn’t manage myself, I would get sick and there would be more financial expenditure, more time spent in hospital, more disturbance in the family, and there would also be a limited opportunity for a person to achieve growth and be more productive. Researcher, University of the Western Cape, South Africa.

The experts suggested that individuals who are around patients should be aware of the details of the disease and how to support it. Furthermore, experts explained that if individuals around patients know details about how to care for the patients, it will increase the positive treatment outcome. In addition, their better knowledge of the disease helps to curb the negative effects of the disease at an individual, family, and societal level, and to avoid stigma and discrimination at a societal level.

### Process dimensions

#### Knowledge and information access of the patient

For HIV patients, having sufficient information about their condition, such as how it gets better, what will happen if it gets worse, and what things should be monitored are essential. Patients should understand the seriousness of their condition and acknowledge, particularly self-care activities that help them to live better and longer. However, as the experts said, most patients are not aware of the seriousness of their problems. Providing comprehensive information by the health care providers will strengthen the ability of PLWHs to self-regulate, which improves the quality of life and motivates the patient more.

They don’t know about some medications and their common side effects because they are not in a position. … What is “lifelong treatment”? Some patients might think that they might be free from the virus [HIV] after one or two years of treatment. When we say lifelong, we mean lifelong. The goal of treatment needs to be clear. So, I think these things should be made clear by the providers. Why I am empathizing with this is because it directly affects their self-management and then treatment outcome, focus should be given to increasing their [patient] awareness. Medical doctor, Ethiopia.

Because of the lack of patient empowerment that prevents them from caring for themselves, even minor ailments, which can be solved at home by themselves, come to overburden the health system.

### Outweighing spiritual beliefs more than medical care

The experts shared their experiences on how some patients prioritize their spiritual beliefs more than taking medications. Some patients perceive that taking ART medications along with their spiritual care will antagonize each other. Hence, most of these patients stop taking drugs and solely rely on their spiritual beliefs. At later times, such patients come to health facilities having complicated health conditions. Other patients sometimes try to link spirituality and treatment together. For instance, some HIV positive patients use holy water (one of the religious services among Orthodox Christians in Ethiopia) with ART medications. Those patients consider their spirituality to help them cope with the severe consequences of disease and poverty. The experts recommended providing health care services linked with their need for spiritual care. The following quote attests to these ideas.

They [patients] may believe that they can be cured by certain religious or spiritual activities. That’s why they just drop the drug and rely on religion, and finally it gets complicated, and they come back to the healthcare professional. And then we ask them why they did this, and they usually say that they were misinformed. Sometimes they are also fed up with medication; … They also want to get rid of HIV. But we told them that it is a lifelong medication… Medical doctor, Ethiopia.

### Self-esteem skills & abilities

Experts suggested that patients should have skills of self-esteem to improve their self-management skills. As per their description, self-esteem will help patients to improve their self-efficacy, disclosure status, adherence to treatment, sexual risk management, and other issues. In general, it contributes to a better health outcome because self-esteem involves goal setting, improving client’s problem-solving skills, and improving resilience or coping strategies through mindfulness and self-compassion. However, most HIV positive patients have low self-confidence and lacks mindfulness, which causes them to be careless about caring for themselves and to have a care plan. Another critical issue impeding self-esteem of patients is stigma and discrimination, which can distract the emotions of patients. Self-esteem is something that will be useful and important in strengthening their ability to manage themselves. The following quote attests to these ideas.

…It is better to work on developing our patients’ confidence. This is one of the common problems that I observe in my routine duty. Most of our HIV patients have low self-esteem, never value themselves, and have a sense of dependency. As a result, it is preferable to devise a strategy for releasing them from this problem. Nurse, Hawassa University, Ethiopia.

### Taking one’s own responsibility and goal setting

Patients should assume the lion’s share of responsibility for improving their health. Our participants stated that patients’ well-being is highly dependent on the ability of patients to manage their chronic disease. Patients who can take ownership or responsibility are aware of and understand what is going on in their lives. And also, they be should be able to set goals and to develop action plans for better health outcomes. Even though goal setting is important for the client, healthcare providers don’t really know how to empower patients in this regard. The following quote attests to these ideas.

I am the master of my destiny. There are a lot of other things around me that interfere with it, but at the end of the day, it is me who wins and wakes up in the morning. Yeah, with that kind of responsibility, it is me who is going to open that bottle and swallow the tablet. I think taking that kind of ownership is very important. Once one understands, then they will be able to take ownership and see the visit to healthcare professionals as their own personal business and plan for achieving the objective they have set for themselves. Researcher, University of the Western Cape, South Africa.

### Social facilitation: sources and types of support

Social support is vital in increasing the quality of life of patients, notably among chronic patients who take medications every day. HIV is a social and economic problem as well. They require more social assistance; nevertheless, stigma and discrimination have a greater detrimental impact on the patient’s social capital. The following are some of the benefits of social support for HIV patients as mentioned by experts:

- It fosters resilience towards social and medical problems of disease, stigma, and discrimination.
- Peer support systems help patients by fostering confidence, solving minor ailments, and getting psychological support.
- Peer groups help to reduce the burden on the health system by sharing some activities.

As it is known in our country [Ethiopia], we do have a limited number of health professionals, so it is difficult to properly counsel every patient, be it on his dietary practice or other healthy lifestyle conditions, so every patient should be linked to adherence supporters and case managers. Nurse, Hawassa University, Ethiopia

#### Self-management practices and recommendations

##### Self-management practices

Most experts believe that the success of ART treatment is strongly contingent on patients’ capacity to maintain good health habits and practices. Besides patient’s lion share, health professionals, family and society have a responsibility for bringing the intended outcome. Most experts believe that health-care providers have a dual responsibility: (1) providing health-care services, and (2) empowering patients to oversee their own care. However, as shown in the preceding sections, HIV patients do not manage themselves well and do not receive an enabling environment from their inner circle, society, or health facility. Most experts advocated for self-management practices to be included in the health system, since the goal of good chronic care should be to boost quality of care through making the patient the hub of his/her own care.

The skills of self-management, besides helping patients to increase their quality of life through boosting positive treatment outcomes, have a positive contribution to reducing load of health facility. One of the experts from South Africa said that they are using self-management as part of the service delivery to reduce the caseload from the health system. The following quotes illustrate the above idea.

In South Africa, in some facilities, due to the high number of patients, we are using self-management as part of care. What we do is equip them [patients] with the skills of self-management. … Those who are stable and have viral suppression come only once a year for symptom screening and to get viral load investigation. For the rest of the time, they must basically care for themselves using their skills of self-management. … Patients need to be empowered to care for themselves since we really don’t have the capacity to have detailed management of patients. Researcher, Stellenbosch University, South Africa.

#### Context-specific and comprehensive self-management

Though most experts agreed that the self-management skills should be comprehensive, it should be based on patient’s context. Adults and children, males, and females, educated and uneducated, the poor and the wealthy, should not receive similar self-management skills. These different categories of people have different characteristics, or they might be in different situations, the type and dose of the problem they are facing might be different. For example, the context and problems that adolescents face before and after starting sexual intercourse cannot be the same and should be managed differently. The following quotes illustrate the above idea.

Adults are often thought to practice self-care. However, many teenagers today are aware of their HIV status and are receiving treatment, so we don’t just have to think about adults only. Moreover, the need and type of self-care before and after conducting sexual intercourse is different since their context varies, so we must make self-care age-appropriate and context specific. Expert, NGOs, Ethiopia.

## Discussion

The study participants identified a variety of individual and community level problems that affects life of people living with HIV, such as poor knowledge and information access, risky behaviors, stigma and discrimination, a lack of commitment, insufficient sources of support, suboptimal goal setting, and low self-efficacy. All problems have a deleterious impact on self-management, treatment outcomes, and the quality of life of PLWH. The professional-centered service delivery in health facilities, lack of family or social support, and inability of health service providers to understand a patient’s needs beyond medical issues, have contributed to the patient’s low self-efficacy and self-management. The study participants claimed that patients have a big stake in improving their own treatment outcomes and quality of life through improved self-management. The experts agreed that self-management practices should be developed and integrated into service delivery to make the patient the center of their own care.

Most HIV patients face several problems, for instance they refuse to accept the reality of being positive and have to live with fear and non-disclosure. They often deny reality and try to avoid disclosing their serostatus out of fear of stigma and discrimination. Existing evidence shows that disclosure increase stigma [6, 34], and being treated differently and isolation even by friends, family, and adverse social reactions, including losing jobs [35]. Such concerns cause HIV-positive people to deny reality and face internalized stigma, which exposes them to higher mental instability and depression, substance use, diminished optimistic outlooks and reduced social support [6, 35, 36]. Furthermore, external stigma has a deleterious effect on attaining positive treatment outcomes by affecting patients’ dreams and goals pertaining to HIV management. In previous studies, HIV-related external stigma was associated with increased perceived goal difficulty and diminished self-efficacy [37, 38]. Stigma, and poor self-confidence were associated with poor self-efficacy [39, 40]. These conditions greatly affect the quality of life of the patient through influencing self-management [41, 42]. Improvement in self-esteem and self-efficacy should be a key focus to HIV care and further research as it will leads to improvement in adherence to care and then to improvement in the quality of life of the patient [43].

The patient’s poor self-management has been exacerbated by professional-centered service delivery in healthcare facilities, the incapacity of health service providers to comprehend a patient’s needs beyond medical concerns, and a lack of family or social support. Improved treatment outcome and better-quality of life among patients needs understanding of the patient’s problem beyond their medical issues [44, 45]. Strong social support reduces HIV-related stigma [6], and health service providers use of self-management improves treatment outcomes of patients [46]. In Ethiopia, a lack of providers’ ability to empower and support HIV patients ultimately affected patients’ self-management skills [47]. Health providers’ inability to empower patients and patients’ lack of social and familial support are circumstances that worsen the disease’s detrimental consequences.

The experts agreed that self-management practices should be developed and integrated into service delivery to make the patient the center of their own care. The IFSMT model showed how multifaceted problems and self-management were affecting the quality of life of the patient and promoted the use of self-management to improve the patient’s quality of life [23]. The findings of the current study and others showed that the success of ART treatment and a good quality of life are strongly contingent on patients’ self-management practices [6, 46]. The patient has a significant role for the successful implementations and effectiveness of self-management [48]. Self-management intervention reduces hospitalizations, frequency of facility visits, and burden that HIV brings on the healthcare system [16–18, 21]. Though self-management is effective, it is seldom practiced in African countries in the case of HIV patients [49, 50]. Furthermore, being considerate of the contexts of patients during self-management development and implementation is crucial, which will help tailor interventions to the specific context of the patient [37, 41, 43, 49–52].

### Limitation of study

The conclusions drawn from this assessment do not reflect what patients are facing or experiencing; rather, they reflect the views of service providers and experts who work in care of HIV patients and other HIV-related issues. On the other hand, the perspective of patients is probably such a big issue that it would have been unwise to try to merge all perspectives into one study, especially considering that this field of research is still in its early stages. Merging the perspectives at later stages is recommended. The other limitation was our inability to include experts from Uganda because of their non-response. Our plan was to include experts from South Africa, Uganda, and Ethiopia, but after the start of the data collection, experts from Uganda did not respond to our invitation to participate in the study. This might have compromised to some extent the strength of our evidence to generalize to the whole African context.

## Conclusion

HIV patients face multifaceted problems on top of their medical issues. The success of medical treatment for HIV is strongly contingent upon patients’ self-management practices and the supportive roles of their family, society, and health service providers. The development and integration of self-management practices into the health care systems of developing countries will benefit patients, their families, and the health system. The service providers also demanded the development, integration, and implementation of self-management into the health care system. Therefore, the researchers of the current study have two recommendations: first, assess patients’ lived experiences and their need for self-management; second, develop comprehensive self-management intervention packages that are tailored to the unique contexts of patients.

## Data Availability

The data is available from the corresponding author on reasonable request.
